# Onsite healthcare worker acceptability and performance of the point-of-care Pima CD4 assay in Dar es Salaam, Tanzania

**DOI:** 10.4102/ajlm.v8i1.740

**Published:** 2019-11-21

**Authors:** Mary E. Schmitz, Karen Chang, Nichole Arnett, Luciana Kohatsu, Ruth Lemwayi, Michael Mwasekaga, John Nkengasong, Omotayo Bolu, Fausta Mosha, Larry Westerman

**Affiliations:** 1 United States Centers for Disease Control and Prevention, Dar es Salaam, United Republic of Tanzania; 2 ASPH/CDC Allan Rosenfield Global Health, Dar es Salaam, United Republic of Tanzania; 3 Division of Global HIV and TB, United States Centers for Disease Control and Prevention, Atlanta, Georgia, United States; 4 African Field Epidemiology Network (AFENET), Dar es Salaam, United Republic of Tanzania; 5 Tanzania Ministry of Health, Community Development, Gender, Elderly and Children, Dar es Salaam, United Republic of Tanzania

**Keywords:** point-of-care, CD4, HIV, user acceptability, microtube

## Abstract

**Background:**

Healthcare workers’ acceptance of and ability to perform point-of-care testing is important for reliable and accurate results. The Alere Pima^™^ CD4 assay (Pima CD4) is the CD4 point-of-care test for HIV management in Tanzania.

**Objectives:**

To evaluate healthcare workers’ acceptance and performance of Pima CD4 testing.

**Methods:**

The study was implemented in five high volume sites in Dar es Salaam, Tanzania, in 2011. Trained healthcare workers performed Pima testing using three whole-blood specimens collected from each patient: venous blood, fingerstick blood directly applied to a Pima cartridge (capillary-direct), and fingerstick blood collected in a microtube (capillary-microtube). Using a semi-structured interview guide, we interviewed 11 healthcare workers about specimen collection methods and Pima CD4 acceptability. Quantitative responses were analysed using descriptive statistics. Open-ended responses were summarised by thematic areas. Pima CD4 results were analysed to determine variation between cadres.

**Results:**

Healthcare workers found Pima CD4 user-friendly and recommended its use in low volume, peripheral facilities. Both venous and capillary-direct blood were considered easy to collect, with venous preferred. Advantages noted with venous and capillary-microtube methods were the ability to retest, perform multiple tests, or delay testing. Pima CD4 results were trusted by the healthcare workers and were in agreement with laboratory Pima testing.

**Conclusion:**

In this point-of-care testing setting, the Pima CD4 assay was accepted by healthcare workers. Both venous and fingerstick capillary blood specimens can be used with Pima CD4, but fingerstick methods may require more intensive training on technique to minimise variation in results and increase acceptability.

## Introduction

Testing for CD4 count was previously a widely used method for determining the timing of antiretroviral therapy (ART) initiation among HIV-positive individuals.^1,2,3^ In 2016, the World Health Organization recommended ART initiation for all HIV-positive adults and children, regardless of CD4 count. However, CD4 testing for baseline and ongoing monitoring of clients on ART where resources allow or if viral load testing is not available is recommended.^4^

In many resource-limited settings, the availability of CD4 testing is challenging due to poor laboratory infrastructure, human resource limitations, instrument maintenance issues, supply chain failures, poor result reporting systems, and insufficient means to ensure quality of testing.^5,6,7,8^ These challenges, coupled with inefficient specimen referral systems, previously resulted in delayed treatment decisions at clinics.^9,10,11^ High attrition rates between HIV diagnosis and ART initiation have been observed with laboratory-based CD4 testing.^9,10,11,12^ Consequently, patients may have progressed to severe or advanced clinical stages of HIV before seeking treatment, dramatically decreasing survival outcomes.^9,10^ While the current Tanzanian guidelines have removed the need for CD4 testing to initiate ART, they still recommend baseline CD4 testing to assess the need for cotrimoxazole preventive therapy and monitoring for any patient with a CD4 level below 350 cells/*μ*L or where viral load testing is not available.^13^ Thus, the testing barriers described will still limit the use of CD4 as a monitoring test, as well as influence wider laboratory monitoring, most importantly HIV viral load.

In addition, the shortage of trained medical personnel remains a barrier in many resource-limited settings attempting to scale up ART programmes.^9,12,14^ Task-shifting from trained laboratorians to other cadres of healthcare workers is a potential solution to the limited access to CD4 testing and other laboratory monitoring for HIV-positive individuals living in remote and isolated areas.^12,14^ The 2016 World Health Organization guidelines also include a recommendation for task-shifting for trained and supervised non-laboratory staff to conduct blood fingersticks for sample collection.^15^ These factors and recommendations for task-shifting underscore the importance of timely laboratory monitoring and user-friendly, point-of-care (POC) testing technologies to allow for decentralised care.

One such technology is the Alere Pima™ CD4 assay (Pima CD4) (Alere Inc., Waltham, Maryland, United States). Point-of-care CD4 assays potentially decrease the turnaround time for the reporting of CD4 results, improving linkage to care and, when CD4 is a criteria, increasing timeliness in ART initiation and treatment switches.^10,11,12,14,16,17,18,19^ Though findings have shown that healthcare workers are able to perform POC testing for patient care,^20^ there is little documentation about the acceptability of the technology among healthcare workers. This study assessed healthcare workers’ acceptance, ease-of-use, and specimen collection preferences for the Pima CD4 assay in the clinical setting in Tanzania. We compared the Pima testers, healthcare workers to laboratorians, to determine agreement with or differences between Pima CD4 results from these cadres. This evaluation was part of a clinical validation of specimen types for the Pima CD4 assay compared to the FACSCalibur platform; the primary study findings have been reported separately.^21^

## Methods

### Ethical considerations

Informed consent was obtained from all participants (patients and healthcare workers) in accordance with the United States Department of Health and Human Services’ 45 CFR 46 Protection of Human Subjects regulation. Ethics approval was obtained from the Tanzania National Institute for Medical Research, the Tanzanian Ministry of Health and Social Welfare and the United States Centers for Disease Control and Prevention, Associate Director of Science (Protocol Number U26GPS002728).

### Study design

Five healthcare sites in Dar es Salaam, Tanzania, providing HIV care and treatment and prevention of mother-to-child transmission services, were purposefully selected for this study. Site selection criteria were high patient volume and proximity to the reference laboratory, National Health Laboratory Quality Assurance Training Center (NHLQATC), in Dar es Salaam, Tanzania. Male and female HIV-positive patients aged between 8 and 65 years attending the selected clinics and requiring a routine CD4 test were eligible for the primary clinical validation study.^21^ The study was implemented over a five-week period in 2011.

Healthcare workers at the study sites collected specimens via three different methods: fingerstick capillary blood directly applied to a Pima CD4 cartridge (capillary-direct), fingerstick capillary blood collected into an ethylenediaminetetraacetic acid (EDTA) microtube (capillary-microtube), and venipuncture blood collected into an EDTA tube (venous). A study trainer certified in phlebotomy trained healthcare workers on fingerstick collection followed by competency testing for all healthcare workers. Peripheral venous whole blood was collected via venipuncture into EDTA vacuum tubes (Becton, Dickinson and Company [BD] Vacutainer, BD Diagnostics, San Jose, California, United States).

Pima CD4 testing was performed at all study sites by healthcare workers using all three specimen collection methods on the day of specimen collection. After Pima CD4 testing at the healthcare site, the EDTA venous whole-blood specimens were transported to the NHLQATC, and Pima CD4 and standard-of-care testing was performed on FACSCalibur (Becton Dickinson, San Jose, California, United States) equipment on either the day of specimen collection or the following day by laboratory technicians. The healthcare workers and NHLQATC laboratory technicians conducting Pima CD4 testing were trained on the Pima CD4 assay and the use and interpretation of quality control materials. Patient participants in the study only received the FACSCalibur CD4 results. Patient participants did not receive the Pima CD4 results because the assay was not approved for use in Tanzania at the time of the study.

### Healthcare worker survey

All healthcare workers involved in blood collection and performing the Pima assay during the study were invited to participate in a voluntary, individual interview at the end of the study implementation. The study coordinator individually interviewed healthcare workers using a semi-structured interview guide. Interviews were conducted in Kiswahili and translated into English for analysis. Questions about each specimen collection method for Pima CD4 testing included: (1) an ease-of-use score on a scale of 1 to 5, with 1 being the easiest and 5 being the most difficult, (2) a ranking of the three sample collection methods in order of preference, and (3) likes and dislikes for each method. The healthcare workers were also surveyed on their experience with the Pima CD4 assay including: (1) the use of Pima CD4 cartridge, (2) performing the Pima CD4 testing, (3) quality control testing, and (4) errors and malfunctions experienced with Pima CD4 testing. The healthcare workers were asked whether they trusted the Pima CD4 results acquired from each of the three specimen collection methods. Finally, healthcare workers were asked open-ended questions about what they liked and disliked about Pima, experiences using the Pima CD4 assay, and recommendations for rollout in Tanzania.

### Data analysis

Quantitative healthcare workers’ survey responses were analysed using Microsoft Excel (Microsoft Corporation, Redmond, Washington, United States) using frequencies and tallies of rankings for mean ease-of-use ranking. Open-ended responses were summarised by thematic areas. For each theme, comparisons between common or divergent views were made.

All Pima CD4 results were analysed in IBM Statistical Package for Social Sciences 21 software (SPSS) (IBM Corporation, Armonk, New York, United States) and Microsoft Excel. Healthcare worker and NHLQATC Pima CD4 results were compared by using scatter plot and best-line analysis with linear regression to estimate the errors by determining coefficient of determination (*R*^2^), slopes, and y-intercepts. In a Bland-Altman analysis, systematic bias and errors between healthcare workers and laboratory technician Pima CD4 testing were estimated.^22^ For Bland-Altman plots, the NHLQATC reference laboratory’s Pima CD4 counts were plotted on the x-axis, and the difference between the healthcare workers’ and NHLQATC laboratorian Pima CD4 counts for each pair of venous blood specimens were plotted on the y-axis. The average differences (bias) and limits of agreement (bias ± 2 standard deviations) were calculated and shown on the Bland-Altman plots.

## Results

The validation study enrolled 1060 patients. Characteristics of study participants; number of invalid tests by specimen type, error type, and site type; number of results per participant by specimen type by site; and performance data compared to the gold-standard FACSCalibur for each specimen type have been reported separately.^21^

Three sample types were collected from each patient: venous blood, capillary-direct, and capillary-microtube. Of the 1060 samples collected by capillary-microtube fingerstick, 25 (2.8%) were of insufficient volume and six (0.6%) were clotted, while for the capillary-direct fingerstick collections, five (0.5%) were of insufficient volume. Thus, during this study, the quality of the specimens collected was acceptable for Pima CD4 testing, with only a few compromised specimens occurring with fingerstick collection. Venous specimens were sufficiently collected and tested for CD4 counts for all patients.^21^

### Healthcare worker survey participants

Of the 20 onsite healthcare workers who supported specimen collection and Pima CD4 testing across the five sites, 11 healthcare workers were interviewed after study enrolment ended to assess user acceptability. The other nine HCWs were unavailable at the time of the interview. The cadre of interviewees included nurse counsellors (5), clinical officers (3), phlebotomists (2), and a laboratory technician (1). Results from healthcare worker interviews were summarised for three topic areas: specimen collection methods, Pima CD4 testing, and recommendations for use of Pima in Tanzania.

### Healthcare worker feedback: Specimen collection methods

Healthcare workers rated capillary-microtube specimens as more difficult to collect when compared to the venous and capillary-direct methods (Table 1). On the ease-of-use ranking, the capillary-microtube method scored an average of 3.0 (95% confidence interval [CI] 2.54–3.46) versus 1.73 (95% CI 1.26–2.19) for the venous method and 1.82 (95% CI 1.18–2.46) for the capillary-direct method. Eight of the 11 healthcare workers interviewed rated the capillary-microtube as the most difficult specimen collection method, with three noting the longer collection time as a disadvantage.

**TABLE 1:** Results of the healthcare worker survey on ease-of-use for collection and testing ranking of specimen collection methods, and trust of Pima CD4 results (*n* = 11), Tanzania, 2011.

Interview no. (site no.)†	Healthcare worker cadre	Ease-of-use rating‡			Method preference ranking	Do you trust the results from each method?††		
		Venous‡	Capillary-direct§	Capillary-microtube§		Venous	Capillary-direct	Capillary-microtube
11 (1)	Nurse Officer, Phlebotomist	1	2	3	V – M – D	Yes	Yes	Yes
05 (1)	Phlebotomist	3	1	4	V – D – M	Yes	Yes	Yes
04 (2)	Nurse Counsellor	1	1	2	V – M – D	Yes	Yes	Yes
03 (2)	Laboratory Technician	1	3	3	V – M – D	Yes	Yes	Yes
01 (3)	Nurse Counsellor	1	3	4	V – D – M	Yes	Yes	Yes†
02 (4)	Nurse Counsellor	3	2	2	V – D – M	Yes	Yes	Yes
06 (4)	Clinical Officer	2	1	2	V – M – D	Yes	‡‡	¶¶
10 (4)	Clinical Officer	1	4	3	V – D – M	Yes	No	Yes
09 (5)	Nurse Counsellor	2	1	3	D – V – M	Yes	Yes	Yes
12 (5)	Nurse Officer, Counsellor	2	1	3	D – V – M	Yes	Yes	Yes
07 (5)	Clinical Officer	2	1	4	D – V – M	Yes	Yes	Yes

D, capillary-direct; M, capillary-microtube; V, venous.

† , There was no interview labelled no. 08 in the data set.

‡ , Ease-of-use rating scale: 1, very easy to 5, very difficult.

§ , Method mean: Venous = 1.73; Capillary-direct = 1.82; Capillary-microtube = 3.00.

¶ , Most preferred ranked first; least preferred ranked last.

†† , Proportions who answered ‘Yes’ to ‘Do you trust the results from each method?’: Venous = 100%; Capillary-direct = 82%; Capillary-microtube = 91%.

‡‡ , ‘Only trust microtube if client is a good bleeder and has good flow’.

¶¶ , ‘Cannot say if I trust the [direct and microtube] results or not’.

The majority (8/11, 73%) of healthcare workers selected the venous specimen as their most preferred method and three (3/11, 27%) selected capillary-direct as their most preferred method (Table 1). The capillary-microtube method was selected as the ‘least preferred method’ by seven (7/11, 64%) of the healthcare workers and the capillary-direct methods was selected by four (4/11, 36%). Interviewees thought the venous method was the easiest and noted that it is the most common method they have been using for other tests performed at the clinic.

### Healthcare worker feedback: Pima CD4 testing

The Pima CD4 assay was viewed positively by all healthcare workers interviewed, with nine specifically noting that it was user-friendly (Table 2). The healthcare workers liked that it was portable, did not require extra reagents, and retained battery life for a long time; however, electricity was still required for charging and could limit the length of time that the Pima CD4 device was operational. They also liked that it required minimal training and could be used by lower cadres. They felt the user guide was helpful; however, several noted that the user guide should be translated into Kiswahili. Two interviewees commented that training should be practical-focused to assess competency. While prompt results were advantageous, several noted the challenge of low machine throughput, particularly for high volume facilities.

**TABLE 2:** Selected qualitative responses from healthcare worker survey, Tanzania, 2011.

Question	Sample of open-ended responses (interview no.)
Specimen collection method: For each method, please tell us what you liked and did not like about that method.	‘Venous easy to collect and good blood flow. Venous is a commonly used method. Microtube is difficult to collect if there is poor flow of blood’. (1)
	‘Venous is very easy and can collect blood samples to test them later. Direct may make people wait for a long time while waiting for their turn to get tested. With microtube, some clients have hard calluses. It is difficult to get enough blood for testing. It takes more time to collect enough sample’. (3)
	‘Venous is the common method we use daily. In case of error, we can repeat the test. Direct is easy but cannot repeat. There is no chance of retesting without pricking the client twice. With microtube, we can retest in case of errors, but difficult collection in case of low blood HB/blood flow’. (4)
	‘With venous, you can collect many samples and perform the test later but it consumes a bit more time as compared to the direct sample. Direct is easy to collect and requires shorter time. But you must be aware of test going on while waiting for results. The client must be present in case you need to retest as there is no opportunity to repeat test without pricking the client again. The microtube consumes a little bit more time but can collect samples, then test later’. (6)
	‘With venous, you can do testing at a later time when more convenient and it is easy to transfer blood into the cartridge. But it can be difficult to get a vein and may take a longer time. Direct is easy if prick it right. Microtube is difficult to get enough sample but you can do testing at a later time’. (9)
Please tell us about your experience using the Pima machine.	‘It is an easy technology. It is a good machine that gives directives like a computer, so it is user-friendly. It gives results promptly, therefore, it is better for client care’. (4)
	‘It is easy and there are no complications. It does not use reagents and gives feedback promptly. It is a portable machine hence it can move from one clinic to another for home-based care. It retains a charge for [*an*] acceptable duration of time’. (5)
	‘It is an easy technology which can be used by even lower cadres. It is good technology and give results quickly. It provides better care to the clients’. (6)
	‘It is user-friendly technology, but it needs electricity for charging. Users should be provided with guidance on how to use it’. (7)
	‘Good technology, however, it should be used in low client volume sites. If it is used in high volume sites, it should be reserved for the very serious clients who need immediate care and CD4 test results. It performs better for 4–5 clients a day’. (11)
Would you recommend that the Pima machine be used in health facilities in Tanzania? Why or why not?	‘Yes, many healthcare workers can use the machine, not only laboratory personnel. It can also be used in any facility’. (6)
	‘Yes, it is user-friendly and it gives results in a short time. Low volume sites, like dispensaries and health centres, should receive the machine. Hospitals can use them as back-ups. However, the manual should be translated into Swahili and training should be done more practically’. (7)
	‘Yes, especially for pregnant women because they need urgent services to prevent MTCT. Health centres and dispensaries should receive the machine because they have no CD4 testing equipment’. (10)
	‘Yes, but in low volume clinics and for special cases to ensure that the machine is not overloaded. All health facilities should use the machine, but should not be overloaded. It should be used only for seriously sick clients and those who need CD4 testing urgently for deciding their treatment course’. (11)
	‘Yes, this will help clients get their CD4 test results as clients always want to know how they respond to the treatment they receive. All health facilities should receive the machine where feasible as all clients and clinicians need to know [*the*] CD4 results of the clients. This machine should start to be used immediately as it can be used anywhere in all facilities since no electricity is needed. Electricity is needed only for charging and the machine can work on battery’. (12)

HB, haemoglobin; MTCT, mother-to-child transmission of HIV; CD4, cluster of differentiation 4.

While most healthcare workers reported that the capillary-direct method was easy to collect and apply to the cartridge, three interviewees cited difficulty applying capillary blood directly to the Pima CD4 cartridge. With both the venous and capillary-microtube specimens, blood was transferred from a tube via a transfer pipette and applied to the Pima CD4 cartridge. Only two (2/11, 18%) of the healthcare workers felt it was difficult to transfer venous blood to the Pima cartridge. Similar to the venous specimen, only one (1/11, 9%) healthcare worker felt that the capillary-microtube was difficult to transfer to the Pima cartridge. Nearly all respondents mentioned that an advantage of the venous and capillary-microtube methods was that they both allowed for retesting or delayed specimen testing if the Pima Analyzer was in use at the time of sample collection. Conversely, healthcare workers noted that the capillary-direct method required the Pima Analyzer to be free immediately after fingersticking, and retesting was only possible by pricking the client a second time. This aspect of the capillary-direct method could increase a client’s waiting time and burden.

Nine (9/11, 82%) of the 11 healthcare workers trusted all Pima CD4 results for all three specimen collection methods (Table 1). All healthcare workers trusted Pima CD4 results with venous specimens. Of the two (2/11, 18%) healthcare workers who expressed some distrust with the Pima CD4 results using fingerstick specimens, one trusted capillary-microtube but not capillary-direct, while the other was indecisive. The former healthcare worker also expressed difficulty obtaining fingerstick samples and a general preference for venous blood as a sample type. Among the nine healthcare workers trusting all three methods, one noted they only trusted capillary-microtube if the client is a ‘good bleeder and has a good flow’.

### Healthcare worker feedback: Recommendations for use of Pima in Tanzania

All interviewees recommended the use of the Pima CD4 assay in Tanzania; however, use should be tailored to specific sites and situations (Table 2). The majority (8/11, 73%) of the healthcare workers recommended targeting use of the Pima CD4 assay to peripheral sites such as health centres and dispensaries or low patient volume sites. The major reason for targeting peripheral sites was due to a general lack of onsite CD4 testing at those levels and delays in acquiring CD4 results from a referral laboratory. Many healthcare workers expressed concerns related to increased patient wait time if the Pima CD4 assay were placed in high volume facilities and used capillary-direct specimens, as the Pima Analyzer can process only one sample at a time. Other healthcare workers mentioned that the Pima CD4 assay could be used at higher-level facilities for targeted populations (such as prevention of mother-to-child transmission of HIV) or used for seriously ill patients in need of urgent CD4 counts. One healthcare worker mentioned that clients would like to receive CD4 results on the same day and this would improve HIV client care.

### Healthcare workers’ performance with Pima CD4 compared to laboratory

The healthcare workers performed 3317 Pima CD4 tests on whole-blood specimens during this study at the five healthcare sites. The capillary-direct method accounted for 1055 of these tests and included 111 (10.5%) invalid tests. The healthcare workers performed 1118 Pima CD4 tests with capillary-microtube specimens with 110 (9.8%) invalid tests and 1144 Pima CD4 tests with venous specimens including 95 (8.3%) invalid tests, while the laboratorian performed 1219 Pima CD4 tests using venous specimens only. The overall Pima CD4 invalid test rate for healthcare workers was 9.5% (316/3317), while the laboratorians invalid test rate was 12.6% (153/1219). For invalid tests where residual samples were sufficient, tests were repeated and the valid repeat result was included in the analysis. Also, six of the valid tests carried out by laboratorians were repeated and the results were also included. A valid test was repeated for a number of reasons such as the wrong Specimen ID number being entered during testing and then repeated with the correct ID or the operator not realising a specimen was already tested.

**FIGURE 1:**
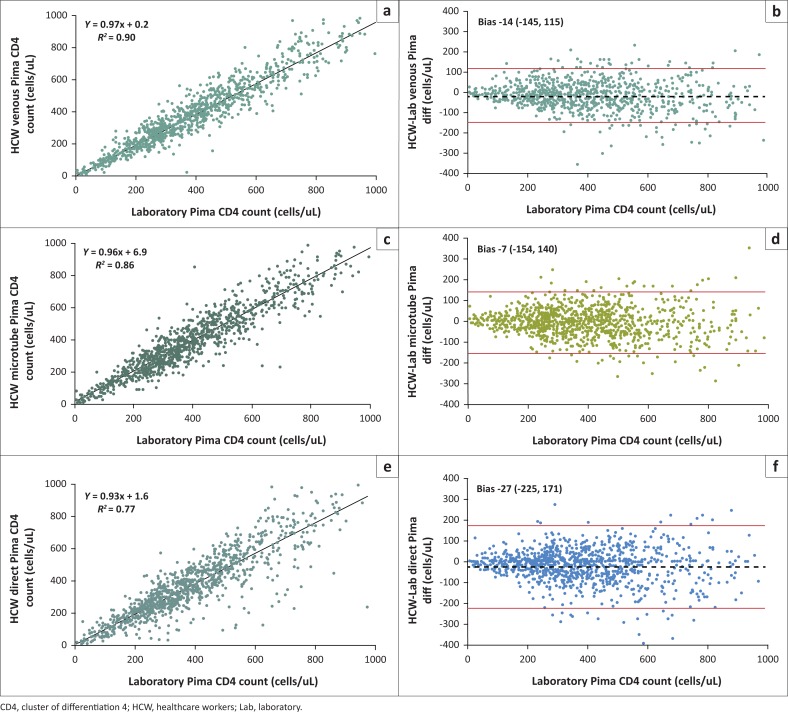
Healthcare worker Pima CD4 results at point-of-care site compared to laboratory Pima CD4 results. Scatter and Bland-Altman plots of healthcare worker Pima CD4 testing with venous blood (a and b), capillary blood collected in ethylenediaminetetraacetic acid microtube (c and d), and capillary blood applied directly to Pima CD4 cartridge (e and f) compared with laboratory Pima CD4 testing with matched venous blood, Tanzania, 2011.

Pima CD4 testing by healthcare workers with fingerstick and venipuncture whole-blood specimens yielded absolute CD4 counts that were in close agreement with paired venous specimens tested by laboratorians with the Pima CD4 assay (Figure 1). The scatter plot analysis of venous blood tested by both healthcare workers and laboratorians, with the Pima CD4 estimated the best-fit line to have a slope of 0.97 and y-intercept of +0.2 with a *R*^2^ value of 0.90. Bland-Altman analysis with the same specimens estimated a bias of -14 cells/uL with acceptable limits of agreement of -145 to +115, indicating good agreement between both cadres, healthcare workers and laboratorians, with venous blood tested on the Pima CD4 assay.

Pima CD4 results by healthcare workers using fingerstick blood, for both the capillary-direct and capillary-microtube methods, when compared to Pima CD4 results by laboratorians using venous blood, also indicated good agreement between the cadres and different specimen types (Figure 1).

When comparing capillary-direct specimen testing results by the healthcare workers to venous blood testing by laboratorians, there was greater variation (more random error) in the Pima CD4 results as indicated by a scatter plot analysis, with a lower *R*^2^ value of 0.77 and Bland-Altman analysis with wider limits of agreement range of 396, (-225 to 171). The *R*^2^ values for the capillary-microtube method (by healthcare workers) was 0.86 and for the venous method (by the laboratorians) was 0.90, while the Bland-Altman limits of agreement ranges were 294 for the capillary-microtube method and 260 for the venous method.

## Discussion

The lack of human resources is a recognised barrier to the continuum of HIV care.^23^ In resource-limited settings, this challenge is evident in all cadres of healthcare workers, including phlebotomists and laboratorians. Task-shifting has been shown to be effective in resource-limited settings and thus may partially address shortages of healthcare workers.^24,25,26^ The innovation of POC testing lies in maximising available resources, so that existing cadres of healthcare workers not only collect specimens but also perform testing for clinical laboratory results.

In studies focusing on the accuracy and reliability of POC testing, the test quality achieved for Pima CD4 assay is reported to be as good as that performed by laboratory-based CD4 assays.^12,17,21,27,28,29,30^ Nevertheless, similar to laboratory-based testing, the use of POC testing can be problematic due to procedural errors during specimen collection, sampling, and testing, lack of clear guidelines for implementation and lack of quality control and quality monitoring.^31,32,33^ Our study assessed the Pima CD4 assay feasibility, acceptability, and validity at the POC or clinical setting with the premise that acceptance and trust of the POC testing is necessary for reliability in testing and accurate results.

All of the healthcare workers interviewed recommended the introduction of the Pima CD4 assay in Tanzania. They expressed that introduction of this POC CD4 assay would improve patient care, which was not a focus of this evaluation; however, other studies have shown improved linkages and earlier ART initiations following the introduction of POC CD4 testing.^10,11,18,19,34^ The healthcare workers in this study appreciated the fact that it required minimal training and could be used across all cadres. Relevant documents should be translated into the dominant local language, in this case Kiswahili, particularly when task-shifting occurs to lower cadres that may not have the technical background and language skills to navigate non-local-language user manuals and training guides.

The Pima CD4 assay has been shown to produce comparable results to standard reference methods of CD4 measurement when using fingerstick capillary and venous blood sample collection methods.^12,14,16,17,20,21,27,30^ While our study had a cadre mix of nurses, phlebotomists, clinical officers, and laboratorians at the site level, a similar study in Kenya compared only lay counsellors to laboratory technicians and found commensurate performance, suggesting that Pima CD4 assays are suitable for use across cadres.^20^ Our study also indicated that various cadres could perform the Pima CD4 assay and achieve similar results as would laboratorians.

POC testing requires that specimen collection and testing be performed within a short period of time. For our study, training for the Pima CD4 assay specimen collection and Pima testing was done simitaneously. As with conventinal laboratory CD4 testing with flow cytometry, the quality POC CD4 test results depends on the quality of the specimen. With training from a certificied phelobotomist, the healthcare workers were able to collect specimens by all three methods in this study. These specimens were of good quality and Pima CD4 results were obtained for almost all study participants.^21^ The healthcare workers were able to correctly perform the Pima CD4 testing on all three specimen types. The Pima invalid test rates from the healthcare workers were similar to those of the trained laboratorians. The invalid test rate for the healthcare workers was 9.5%. As the healthcare workers become more familiar to performing the assay, we believe this invalid test rate will be lower.

Several respondents expressed greater confidence in venous and capillary-microtube results as compared to capillary-direct; however, only one said they did not trust capillary-direct results. This greater confidence with the venous and capillary-microtube methods corroborates healthcare workers’ expression of difficulty in applying the sample directly to the Pima cartridge. Relative to the venous and capillary-microtube methods, capillary-direct had a slightly higher frequency of invalid Pima tests and more results that were variable as evidenced by the lower *R*^2^ and wider limit of agreement range as compared to the venous and capillary-microtube methods. A similar increase in invalid test rates and variability for capillary samples compared to venous samples has been reported in other Pima evaluations.^16,17,21,35^ The invalid Pima tests were primarily due to problems with specimen integrity, improper filling of blood in the cartridge, incorrect handling of the cartridge, or abortion of the assay process by the operator.

In this study, client perspectives on the Pima CD4 assay and the preferred sample collection methods were not obtained. In other studies, clients shared similar preferences for venous blood collection to avoid multiple fingersticks or stated that fingersticks were more uncomfortable than venipuncture.^27,30^ However, a South African study found multiple fingersticks were acceptable by the clients.^36^

### Limitations

This study has several limitations. Participating healthcare workers were few (only 20) and among those, only 11 were available for an interview, which was only about half of an already small pool of healthcare workers. However, to ensure participation in interviews was voluntary, healthcare workers had the opportunity to decline participation. As a result, our sample of healthcare workers included only those who had strong opinions about the Pima assay and were willing to participate. In addition, the study sites were not randomly selected, introducing potential selection bias. All sites were high volume urban facilities where phlebotomy is performed on upwards of 100 clients per day. Although the venous and capillary-direct methods were both seen as easier to use than capillary-microtube, the overall preference for venipuncture may have been influenced by the high volume of phlebotomy at all the sites. Therefore, the user group was not representative of potential Pima users in rural, peripheral facilities. All healthcare workers were interviewed separately; however, it is possible that they built consensus among themselves through shared experiences and training, which might have influenced their individual preferences. Another limitation is that the questionnaire used had not been validated for this type of study. In addition, the questionnaire did not specifically seek views on the length of time to collect Pima samples, run the test, or the overall work burden. Furthermore, due to the small sample size and the qualitative nature of the study interviews, we were not able to conduct multivariate quantitative analysis to adjust for confounders and identify factors associated with outcomes, such as healthcare worker cadre. Lastly, there was a significant delay between study implementation and publication, approximately 8 years; thus, conclusions should be interpreted with awareness that contextual factors may have changed since the time of study implementation.

### Conclusion

Overall, healthcare workers found the Pima™ CD4 assay to be user-friendly and recommended its use for HIV care. The healthcare workers were able to successfully collect venous and fingerstick specimens and perform POC Pima CD4 testing, with results comparable to reference laboratory Pima results. Many healthcare workers noted that collecting whole blood in an EDTA tube, either by venipuncture or by fingerstick, was advantageous as it allowed for specimens to be tested at a later time or to be retested without going back to the patient for another specimen. Despite the strong caveat of this study, its findings suggest that venous or capillary-microtube specimen collection may be more suitable for POC testing workflow with Pima CD4 testing, rather than direct application of fingerstick capillary blood to a Pima CD4 cartridge. Although healthcare workers recognised that the capillary-microtube specimens provided similar benefit to the venous method for retesting, more training and familiarisation with this method of capillary blood collection may be required to increase healthcare worker acceptability. Future studies could consider collecting a larger, more representative sample of healthcare worker feedback, as well as client preferences for sample collection methods for Pima and other devices with options for capillary and venous blood use. In addition, our findings regarding preference for venous or capillary-microtube specimen collection may be applicable to other types of POC testing.
